# Childhood Maltreatment and Mobile Phone Addiction Among Chinese Adolescents: Loneliness as a Mediator and Self-Control as a Moderator

**DOI:** 10.3389/fpsyg.2020.00813

**Published:** 2020-05-12

**Authors:** Shutao Ma, Yuhuai Huang, Yankun Ma

**Affiliations:** School of Education, Guangzhou University, Guangzhou, China

**Keywords:** childhood maltreatment, mobile phone addiction (MPA), loneliness, self-control, adolescents

## Abstract

Previous studies have found that childhood maltreatment is an important risk predictor of adolescent mobile phone addiction (MPA). However, little is known about the mediating and moderating mechanisms underlying this association. Grounded in the Basic Psychological Needs Theory and the organism-environment interaction model, this study examined the mediating effect of loneliness and the moderating effect of self-control in the relationship between childhood maltreatment and adolescent MPA. A total of 981 Chinese adolescents (Mage = 13.68 years, *SD* = 0.92) completed measures regarding childhood maltreatment, MPA, loneliness, and self-control. After controlling for participants’ demographic variables, loneliness partially mediated the relation between childhood maltreatment and adolescent MPA and this indirect path was moderated by self-control. Specifically, the effect of loneliness on MPA was stronger for adolescents with lower self-control than for those with higher self-control. Our research provides additional evidence for the negative association between childhood maltreatment and MPA.

## Introduction

By June 2019, the number of Internet users in China had reached 854 million, among which the proportion of mobile phone users was 99.1%, with teenagers accounting for 16.9% of the total mobile phone subscribers (China Internet Network Information Center, 2019). The report clearly demonstrates that the connection between personal life and mobile phone use is becoming increasingly stronger. Meanwhile, while recognizing the great convenience brought by mobile phones, the problems they cause should not be overlooked. Mobile phone addiction (MPA) refers to a strong desire to engage in mobile phone use, leading to obvious physical and/or psychological maladjustment in individuals (Liu et al., 2018). Both Internet addiction and MPA are addictive behaviors related to excessive use and dependence on a specific medium (Chiu et al., 2013). Previous studies have shown that Internet addiction primarily involves online video game addiction. Given the fact that mobile phones are portable and use smart technology, mobile addicts are addicted not only to mobile games, but also to social network services. This enables individuals to experience more pleasure and immersion from mobile phones (Jeong et al., 2016). A large number of studies have shown that MPA could exert serious negative effects on academic achievement, relationships, psychological health, and sleep quality (Elhai et al., 2017; Han et al., 2017). Therefore, an active exploration of factors affecting MPA and their internal mechanisms is not only of great practical significance for interventions targeting adolescent MPA, but also of great guiding value in order to maintain a healthy life.

Childhood maltreatment involves an act committed by an individual who has the duty to nourish, support, and supervise a child in the child-rearing process, including physical abuse, emotional abuse, physical neglect, emotional neglect, and sexual abuse, which is sufficient to cause actual or potential harm to the child’s health, survival, growth, development, and dignity (Wang et al., 2019). An investigation revealed that a third of Chinese adolescents experienced physical abuse, a fifth experienced emotional abuse, and two-fifths were neglected in childhood (Fang et al., 2015). A series of studies showed that childhood maltreatment is an important risk factor of adolescent problematic behavior (e.g., MPA; Hagborg et al., 2017; Kwak et al., 2018). For instance, Kwak et al. (2018) found that adverse family environments, such as physical neglect and emotional neglect, were positively associated with adolescent MPA. A study by Hagborg et al. (2017) has shown that emotional maltreatment can reduce the emotional experience of adolescents and thus increase their risk of developing problem behaviors. Furthermore, different types of childhood maltreatment will exert adverse effects on the physical and mental development of adolescents. Therefore, it is necessary to explore the influence of overall childhood maltreatment on adolescent problematic behavior (e.g., MPA). However, previous studies mostly focused on the direct relationship between childhood maltreatment and adolescent MPA, while the influence mechanism (e.g., how childhood maltreatment leads to adolescent MPA) was rarely studied. To fill this gap, this study aimed to investigate two questions: (a) whether loneliness mediated the relation between childhood maltreatment and adolescent MPA, and (b) whether this indirect path was moderated by self-control.

### The Mediating Role of Loneliness

Although the relationship between childhood maltreatment and adolescent MPA has been well confirmed, the mediating and moderating mechanisms underlying this relation are worth further studying. Loneliness is defined as a type of negative and diffuse psychological state resulting from individuals’ perceived dissatisfaction with others and with maintaining interpersonal relationships (Asher et al., 1984). On one hand, childhood maltreatment is closely related to adolescent loneliness and positively associated with children’s loneliness as demonstrated by Shevlin et al. (2015). Another study investigated a group of patients with major depression, finding that their sense of loneliness mainly originated from their history of childhood physical abuse and neglect (Kao et al., 2014). On the other hand, the relationship between loneliness and adolescent MPA was also effectively established (Jin and Park, 2012), with a previous study exploring the factors affecting MPA among middle school students establishing that loneliness had a significant impact on MPA (Lee et al., 2013).

According to the Basic Psychological Needs Theory (Ryan and Deci, 2000), if individuals are in an environment where their basic psychological needs cannot be satisfied, such dissatisfaction might lead to a strong cognitive dissonance (e.g., loneliness). Meanwhile, individuals might find other ways to compensate for the unmet needs (e.g., using mobile phones). Overall, adolescents who experienced abuse and neglect were more likely to experience loneliness due to unmet basic psychological needs (Shevlin et al., 2015). In order to alleviate loneliness, adolescents might obtain friendship and social support through mobile phone use, which in turn boosts the risk of MPA (Lee et al., 2013). Specifically, loneliness might be an important mediator in the link between childhood maltreatment and adolescent MPA.

### The Moderating Role of Self-Control

Despite the fact that childhood maltreatment can increase the probability of MPA in adolescents, not all teenagers who experience childhood maltreatment suffer from loneliness and further develop MPA. Therefore, it is imperative to investigate whether the relationship between childhood maltreatment and adolescent MPA might be moderated by other factors. Self-control refers to the process whereby individuals make efforts to shift their responses to external stimulation instead of trying to change the external reality, in order to consciously control their behaviors (Hirschi and Gottfredson, 1990). Self-control is conceptualized into an impulse system and a self-control system in accordance with the dual-system model (Dvorak et al., 2011). Individuals with high scores on the impulse system tend to show a high interest in novel things, and react more simply and quickly, but have a lower ability to resist temptation. Those with high scores on the self-control system think and plan before responding to behaviors and are more resistant to temptation. Therefore, different levels of self-control influence problematic behavior in different degrees. A study suggested that adolescents with low self-control tended to show the characteristics of impulsiveness, risk-taking, and a preference for fresh physical and mental stimulation, as well as for simple rather than complex tasks (Kim and Davis, 2009). Moreover, Mei et al. (2016) reported that adolescents with higher self-control were less likely to engage in addictive behavior compared to those with lower self-control.

The organism-environment interaction model (Lerner et al., 2006) states that the same adverse environmental factors (e.g., childhood maltreatment) do not affect individuals equally; the interaction between individual traits (e.g., self-control) and environmental aspects determines the outcome of an individual’s development (e.g., MPA). A great deal of studies indicate that self-control plays a significant moderating role between an adverse environment and adolescent problem behaviors (Li et al., 2013; Park et al., 2014; Tao et al., 2016). For instance, self-control negatively predicted Internet addiction, with a low level of self-control intensifying the adverse effect of social relationships on Internet addiction. Compared to those with lower self-control, individuals with higher self-control presented a lower probability of problematic Internet use (Park et al., 2014). In addition, Li et al. (2013) demonstrated that self-control was an important protective factor between school connectedness and problematic Internet use.

Based on previous studies on mediating and moderating models, if the relation between childhood maltreatment and MPA were mediated by loneliness and moderated by self-control, the mediating effect of loneliness could be moderated by self-control. As a result, a moderated mediation model could be structured with loneliness as a mediator and self-control as a moderator in the relationship between childhood maltreatment and MPA. On the basis of the above evidence and analysis, we proposed the following hypotheses:

***Hypothesis 1***: Loneliness mediates the relationship between childhood maltreatment and adolescent MPA.

***Hypothesis 2***: The direct and/or indirect association between childhood maltreatment and MPA through loneliness varies as a function of self-control. Specifically, this direct and/or indirect relation will be weaker for adolescents with high levels of self-control.

### The Present Study

**FIGURE 1:**
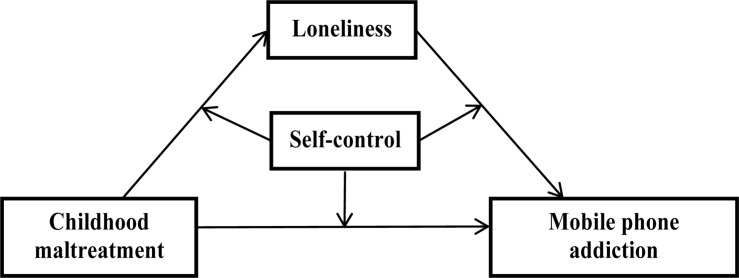
A moderated mediation model of childhood maltreatment, loneliness, self-control, and adolescent mobile phone addiction.

Based on the Basic Psychological Needs Theory and the organism-environment interaction model, this study investigated whether loneliness mediated the relation between childhood maltreatment and adolescent MPA, and whether this direct and/or indirect link was moderated by self-control. Figure 1 shows the moderated mediation model.

## Methods

### Participants

In this study, data were collected from two public junior high schools randomly selected in Guangdong province, China. Our survey was conducted on a class basis, and a total of 1,018 students agreed to participate. As the participants were teenagers, the written consent of participants and their guardians was obtained before the questionnaires were distributed. After data were collected, the contents of 37 questionnaires were found to be incomplete; therefore, only 981 valid questionnaires were analyzed. The sample consisted of 981 students from 7th to 9th grade, identified through cluster random sampling. There were 476 girls and 505 boys, with a mean age of 13.68 years (*SD* = 0.92).

### Procedure

The written consent of participants, their guardians, and teachers included in the study was obtained before the survey was officially started. Furthermore, the questionnaires were distributed, and the data were collected by well-trained graduate students majoring in Psychology. All the participants were informed that participation in the study was entirely voluntary and that they were free to withdraw at any time. Meanwhile, names were not required on the questionnaires, the strict confidentiality of students’ responses was assured, and the data collected were used for academic research only. In addition, our testing material and survey procedures were approved by the Ethics in Human Research Committee of the School of Education, Guangzhou University.

### Measures

#### Childhood Maltreatment

The Childhood Trauma Questionnaire (CTQ; Bernstein et al., 2003) was used to assess childhood maltreatment. It is a 28-item measure that comprises five subscales: emotional abuse (e.g., “At the time, someone in my family often said insulting words to me”), physical abuse (e.g., “At the time, someone in my family beat me so hard I had to go to hospital”), sexual abuse (e.g., “At the time, someone in my family tried to get me to do or watch something sexual”), emotional neglect (e.g., “At the time, I didn’t feel loved by my family”), and physical neglect (e.g., “At the time, no one took care of me”). Participants answered the questions on a 5-point scale (1 = *never true*, 5 = *always true*). Responses across the 28 items were averaged, with higher scores representing higher levels of childhood maltreatment. Previous studies reported that the Chinese version of the CTQ showed good validity and reliability with adolescents (Wang et al., 2019). In this study, Cronbach’s alpha for the scale was 0.79.

#### Self-Control

The 19-item Self-Control Scale (SCS; Tangney et al., 2004) was used to measure adolescents’ level of self-control. It includes four subscales: deliberate and non-impulsive action (e.g., “I am easy to lose temper”), healthy habits (e.g., “It’s difficult for me to get rid of bad habits”), resistance to temptation (e.g., “Sometimes I couldn’t help doing things that I knew were wrong”), and work ethic (e.g., “I have difficulties to concentrate”). Participants rated each item on a 5-point scale (1 = *strongly disagree*, to 5 = *strongly agree*). Scores of the 19 items were averaged; higher scores represented higher levels of self-control. This scale has demonstrated good reliability and validity in Chinese adolescents in previous research (Jiang and Zhao, 2016). Cronbach’s alpha was 0.82 in the present study.

#### Loneliness

Adolescents rated their subjective feelings of loneliness and social isolation using the Children’s Loneliness Scale (CLS; Asher et al., 1984). It consists of 24 items, with 16 items focusing on loneliness and social dissatisfaction (e.g., “No one wants to play with me”), while the other 8 items are about hobbies and interests (e.g., “I like drawing pictures”). Each item was rated on a 5-point scale from 1 (*never*) to 5 (*always*). The mean of the 16 items was calculated, with higher scores reflecting a higher level of loneliness. The questionnaire presented good reliability in previous research (Tan et al., 2016). In the current study, Cronbach’s alpha for the scale was 0.75.

#### MPA

Adolescents’ MPA was assessed with the widely used Mobile Phone Addiction Index (MPAI) developed by Leung (2008). It contains 17 items assessing the extent of mobile phone use, including control craving (e.g., “You find it difficult to turn off your phone”), anxiety and feeling lost (e.g., “You’ll feel anxious if your phone has been off for a while”), withdrawal and escape (e.g., “You talk to people on your mobile phone when you feel lonely”), and productivity loss (e.g., “You would rather play with your mobile phone than deal with emergencies”). Items are rated on a 5-point scale (1 = *completely disagree*, 5 = *completely agree*), with higher values reflecting more severe addiction symptoms. Previous studies have confirmed that the questionnaire is highly reliable and suitable for the Chinese population (Lian et al., 2016; Liu et al., 2018). In the present study, Cronbach’s alpha was 0.85.

### Statistical Analysis

First, we calculated descriptive statistics and performed correlation analyses. Second, we used the MacKinnon’s (2008) four-step procedure to examine the mediation model and followed Muller et al.’s (2005) guidelines to test the moderated mediation model. Besides, previous studies have showed that gender differences, as well as age differences, exist in MPA (Chiu et al., 2013; Jia et al., 2014), therefore, we considered gender and age as control variables in this study.

## Results

### Preliminary Analyses

Table 1 shows the descriptive statistics and correlation matrix for the study variables. As expected, childhood maltreatment was positively correlated with loneliness, and both childhood maltreatment and loneliness were positively correlated with MPA. Self-control, on the other hand, was negatively correlated with childhood maltreatment, loneliness, and MPA.

**TABLE 1:** Means, standard deviations, and correlations of main study variables.

Variables	Mean	Standard deviation	1	2	3	4
1. Childhood maltreatment	2.16	0.41	1.00			
2. Self-control	2.49	0.58	–0.13**	1.00		
3. Loneliness	2.51	0.44	0.52**	–0.13**	1.00	
4. Mobile phone addiction	2.05	0.57	0.38**	–0.34**	0.33**	1.00

**p < 0.01.

### Mediation Model

To test the Hypothesis 1 (Table 2), we followed MacKinnon’s (2008) four-step procedure to establish mediation effect, which requires (1) a significant association between childhood maltreatment and MPA, (2) a significant association between childhood maltreatment and loneliness, (3) a significant association between loneliness and MPA after controlling for childhood maltreatment, and (4) a significant coefficient for the indirect path between childhood maltreatment and MPA via loneliness.

**TABLE 2:** Mediation effect of childhood maltreatment on MPA.

Predictors	Model 1 (criterion: MPA)			Model 2 (criterion: loneliness)			Model 3 (criterion: MPA)		
	*b*	*SE*	*T*	*B*	*SE*	*T*	*b*	*SE*	*T*
Childhood maltreatment	0.49	0.04	12.40***	0.53	0.03	18.07***	0.35	0.02	7.90***
Loneliness							0.24	0.05	5.72***
**Control variables**									
Gender	0.07	0.03	2.07*	0.06	0.02	2.16*	0.05	0.032	1.66
Age	0.04	0.02	2.31*	–0.02	0.01	–0.68	0.04	0.027	2.53*
*R*^2^	0.24			0.30			0.27		
*F*	45.22***			60.83***			40.393***		

N = 981. Beta values are standardized coefficients. Gender was dummy-coded such that 0, female and 1, male. MPA, mobile phone addiction; SE, standard error. *p < 0.05, ***p < 0.001.

Table 2 shows that after gender and age were controlled for, childhood maltreatment had a significant direct effect on loneliness (*b* = 0.53, *p* < 0.001) and adolescent MPA (*b* = 0.49, *p* < 0.001). In addition, loneliness had a direct effect on adolescent MPA (*b* = 0.24, *p* < 0.001). Furthermore, these results demonstrated that the relationship between childhood maltreatment and adolescent MPA was partially mediated by loneliness. In the end, we used the bias-corrected percentile bootstrap method to examine the mediation model. By random sampling, 5,000 bootstrapping samples were created from the original database. The indirect influence of loneliness was 0.13 with a 95% confidence interval (CI) of [0.07, 0.19], and the mediating effect accounted for 20% of the total effect in the link between childhood maltreatment and MPA.

### Moderated Mediation Model

In order to test Hypothesis 2, we used the approach suggested by Muller et al. (2005) (Table 3). In the first model, we estimated the moderating effect of self-control on the influence of childhood maltreatment on MPA. In the second model, we estimated the moderating effect of self-control on the impact of childhood maltreatment on loneliness with MPA. In the third model, we estimated the moderating effect of self-control on both the partial effect of loneliness on MPA and the residual effect of childhood maltreatment on MPA.

**TABLE 3:** Moderated mediation effects of childhood maltreatment on Mobile Phone Addiction (MPA).

	Model 1 (criterion: MPA)			Model 2 (criterion: loneliness)			Model 3 (criterion: MPA)		
	*B*	*SE*	*t*	*b*	*SE*	*t*	*b*	*SE*	*t*
Childhood maltreatment X	0.49	0.04	12.40^∗∗∗^	0.53	0.03	18.07^∗∗∗^	0.35	0.02	7.90^∗∗∗^
Self-control MO	–0.30	0.03	–10.89^∗∗∗^	–0.03	0.02	–1.28	–0.30	0.05	–10.99^∗∗∗^
X × MO	–0.03	0.04	–0.79	–0.04	0.03	–1.38	0.07	0.03	1.24
Loneliness ME							0.24	0.05	5.72^∗∗∗^
ME × MO							–0.12	0.04	−2.17^∗^
**Control Variables**									
Gender	0.07	0.03	2.07^∗^	0.06	0.02	2.16^∗^	0.05	0.03	1.66
Age	0.04	0.02	2.31^∗^	–0.02	0.01	–0.68	0.04	0.02	2.53^∗^
*R*^2^	0.24			0.30			0.27		
*F*	45.22^∗∗∗^			60.83^∗∗∗^			40.39^∗∗∗^		

N = 981. Beta values are standardized coefficients. Gender was dummy-coded such that 0, female and 1, male. *p < 0.05, ***p < 0.001.

The moderated mediation analyses using loneliness as a mediator and self-control as a moderator in the relationship between childhood maltreatment and MPA after gender and age were controlled for are presented in Table 3. Given that the mediation effect was already illustrated above, here we report only the interaction effects between the variables contained in the hypothesis model. As can be seen in Table 3, the result of the interaction effect between childhood maltreatment and self-control (X × MO) indicated that the effect of childhood maltreatment on loneliness (*b* = −0.04, *p* > 0.05) and MPA (*b* = −0.03, *p* > 0.05) was not moderated by self-control, while the interaction effect (*b* = −0.12, *p* < 0.05) between loneliness and self-control (ME × MO) was significant, demonstrating that the effect of loneliness on MPA depended on the moderator.

**FIGURE 2:**
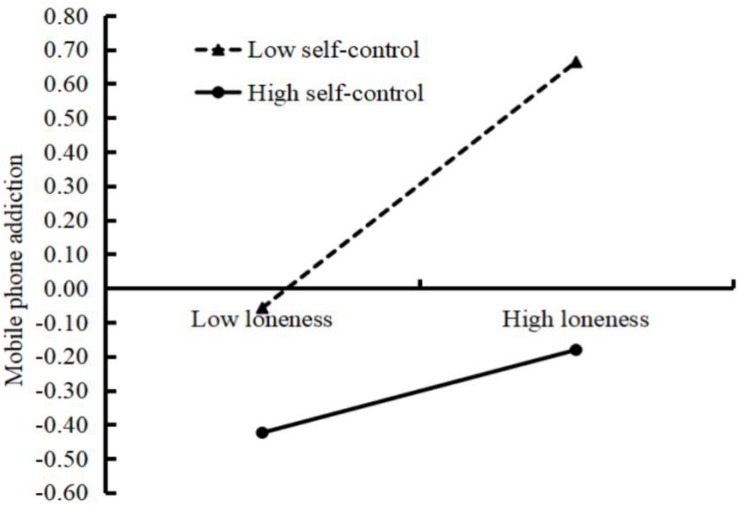
Conditional effect of mobile phone addiction as a function of loneliness and self-control. Functions are graphed for two levels of self-control: 1 standard deviation above the mean and 1 standard deviation below the mean.

To better understand the moderating effect of self-control, the plot of the relation between loneliness and MPA at two levels of self-control (1 SD below the mean and 1 SD above it) is presented in Figure 2. A simple slope test showed that, for adolescents with low self-control, loneliness was positively associated with MPA (*b* = 0.28, *SE* = 0.05, *t* = 5.21, *p* < 0.001, 95% CI = [0.17, 0.39]), while the effect was much weaker for adolescents with high self-control (*b* = 0.15, *SE* = 0.05, *t* = 2.91, *p* < 0.01, 95% CI = [0.05, 0.25]).

## Discussion

The present study utilized a moderated mediation model to examine the mediating and moderating mechanisms in the association between childhood maltreatment and adolescent MPA. The results indicated that the detrimental effect of childhood maltreatment on adolescent MPA was explained in part by loneliness. Moreover, this indirect link was stronger for individuals with lower self-control than for those with higher self-control. These findings provide a more detailed understanding of how and when childhood maltreatment is related to adolescent MPA.

Previous studies have identified a certain association between childhood maltreatment and MPA in adolescents (Zvara et al., 2016; Roche et al., 2018). This study showed that loneliness could significantly mediate the relation between childhood maltreatment and adolescent MPA. In other words, childhood maltreatment could intensify adolescents’ loneliness, which in turn promotes their risk of MPA. This finding is similar to the notion that loneliness, as a negative psychological state, is caused by the adverse external environment and is closely related to adolescents’ addictive behavior (He et al., 2014). In addition, this study provides support for the Basic Psychological Needs Theory. The satisfaction of psychological needs is the basic motivation of individuals’ behavior. If individuals’ basic psychological needs cannot be satisfied, a strong desire to meet these needs will arise, thus forcing individuals to turn to other contexts where such needs can be met (Ryan and Deci, 2000). Specifically, an adverse environment leads to cognitive biases in adolescents, which results in more negative emotions and fewer social relationships. To address the need to feel loved and connected, adolescents make use of the virtual world to cope with loneliness and gain social support. Social networks are extremely open and inclusive (Shevlin et al., 2015), and mobile phones are one of the most common tools for online social engagement (Lee et al., 2013).

Furthermore, we found that self-control moderated the direct association between loneliness and MPA. Specifically, self-control weakened the link between childhood maltreatment, loneliness, and MPA through the direct relation between loneliness and MPA. This result is similar to previous studies indicating that self-control, as a positive individual trait, not only can directly predict problematic behaviors but can also alleviate the influence of negative mental states (e.g., loneliness) on problematic behaviors (e.g., MPA; Li et al., 2013). Individuals with high self-control are able to control their emotions (e.g., loneliness) and external behaviors (e.g., MPA) in accordance with social standards or their own will (Hirschi and Gottfredson, 1990). For instance, Park et al. (2014) found that self-control moderated the indirect effect of peer relationship on problematic Internet use via self-esteem. Similarly, a previous study reported that the negative link between loneliness and adolescent Internet addiction was substantially stronger among adolescents with low self-control than in those with high self-control (Özdemir et al., 2014). In addition, this study has further confirmed and expanded on the organism-environment interaction model by showing that individuals with high self-control were able to better moderate their negative emotions and cognitive biases, and even those who had experienced parental abuse and neglect were less likely to become addicted to mobile phones due to loneliness exerted by these traumas if their self-control was high. Specifically, self-control alleviated the negative impact that loneliness, an adverse cognition, had on adolescent MPA. For adolescents with low self-control, loneliness was a significant predictor of MPA; among those with high self-control, loneliness also had a predictive effect on MPA, the effect, however, being much weaker.

In addition, this study found that the direct effect of both paths from childhood maltreatment to loneliness and MPA was not moderated by self-control. A possible reason is that childhood maltreatment has a strong and lasting impact on adolescents, with a profound negative effect on their development (Johnson et al., 2017). Previous research found that in retrospective questionnaires, adolescents with problematic behaviors have difficulty forgetting their unfortunate experiences in childhood (Liebschutz et al., 2018). A 1 year longitudinal study showed that a negative parent-child relationship experienced by an individual in childhood could have a profound and lasting adverse effect on future behaviors (Ko et al., 2015). Serving as a powerful influencing factor, self-control, however, does not easily moderate the relationship between childhood maltreatment and MPA. Meanwhile, another study found that negative parenting was negatively correlated with self-control (Van Prooijen et al., 2018). In a survey of adolescents with traumatic childhoods, it was found that adverse family environment and parenting style were the main causes of poor self-control (Walters, 2018). The effect of long-term negative family environment on loneliness is not effectively moderated by self-control (Betts and Rotenberg, 2010). A research by Liu et al. (2017) revealed that loneliness has strong stability and durability, and is difficult to alter through positive psychological traits such as self-control. In conclusion, adolescents who have experienced childhood maltreatment, even though they are no longer abused, can still suffer from problematic behavior and cognition, and this influence is difficult to moderate through self-control.

### Limitations and Implications

The limitations of this study are as follows. First, its cross-sectional design does not allow the inference of causal relationships. Future research may need to reveal causality using longitudinal or experimental designs. Second, as the generalization of our results from this small sample of Chinese adolescents is problematic, future research needs to examine the model in diverse and large populations. Third, the relation between childhood maltreatment and MPA could be affected by various factors; however, only two covariates (gender and age) were contained in the hypothesis model. Including insufficient covariates may limit the model application; thus, more covariates should be considered in future research.

Despite the limitations above, we further explored the mediating and moderating factors underlying the association between childhood maltreatment and MPA on the basis of previous studies, and revealed, for the first time, the mediating role of loneliness and the moderating role of self-control. Besides, this study is of great significance for the prevention and management of adolescent MPA. Our findings indicated that childhood maltreatment was closely related to loneliness, which had a strong connection with MPA. Therefore, we suggest that parents could decrease their children’s loneliness by improving their parenting ability and providing more emotional care, which in turn could reduce the risk of adolescent MPA. In addition, the results of this study also confirmed the moderating role of self-control in the association between loneliness and adolescent MPA, indicating that self-control alleviated the negative effect of loneliness on MPA. Specifically, for the high self-control individuals, the negative impact of loneliness on MPA was significantly moderated. Based on the power model of self-control, self-control as an ability can be effectively improved through regular training (Baumeister and Tice, 2007). Therefore, we suggest that parents should promote the self-control training of their children in order to lower their risk of developing MPA.

## Data Availability Statement

The raw data supporting the conclusions of this article will be made available by the authors, without undue reservation.

## Ethics Statement

The studies involving human participants were reviewed and approved by The Research Ethics Committee of the School of Education, Guangzhou University. The participants and their legal guardians provided their written informed consent to participate in this study.

## Author Contributions

SM and YM conceived and designed the research, performed the research, and wrote the manuscript. SM collected and analyzed the data. SM, YH, and YM revised the manuscript. YM and SM approved the final version to be published.

## Conflict of Interest

The authors declare that the research was conducted in the absence of any commercial or financial relationships that could be construed as a potential conflict of interest.
